# Supplementary material for: Novel Mitochondrial Gene Rearrangement and Intergenic Regions Exist in the Mitochondrial Genomes from Four Newly Established Families of Praying Mantises (Insecta: Mantodea)
Source: Insects. 2022 Jun 21;13(7):564. doi: 10.3390/insects13070564 (PMC9320148; doi:10.3390/insects13070564)

A. *Carrikerella* sp.

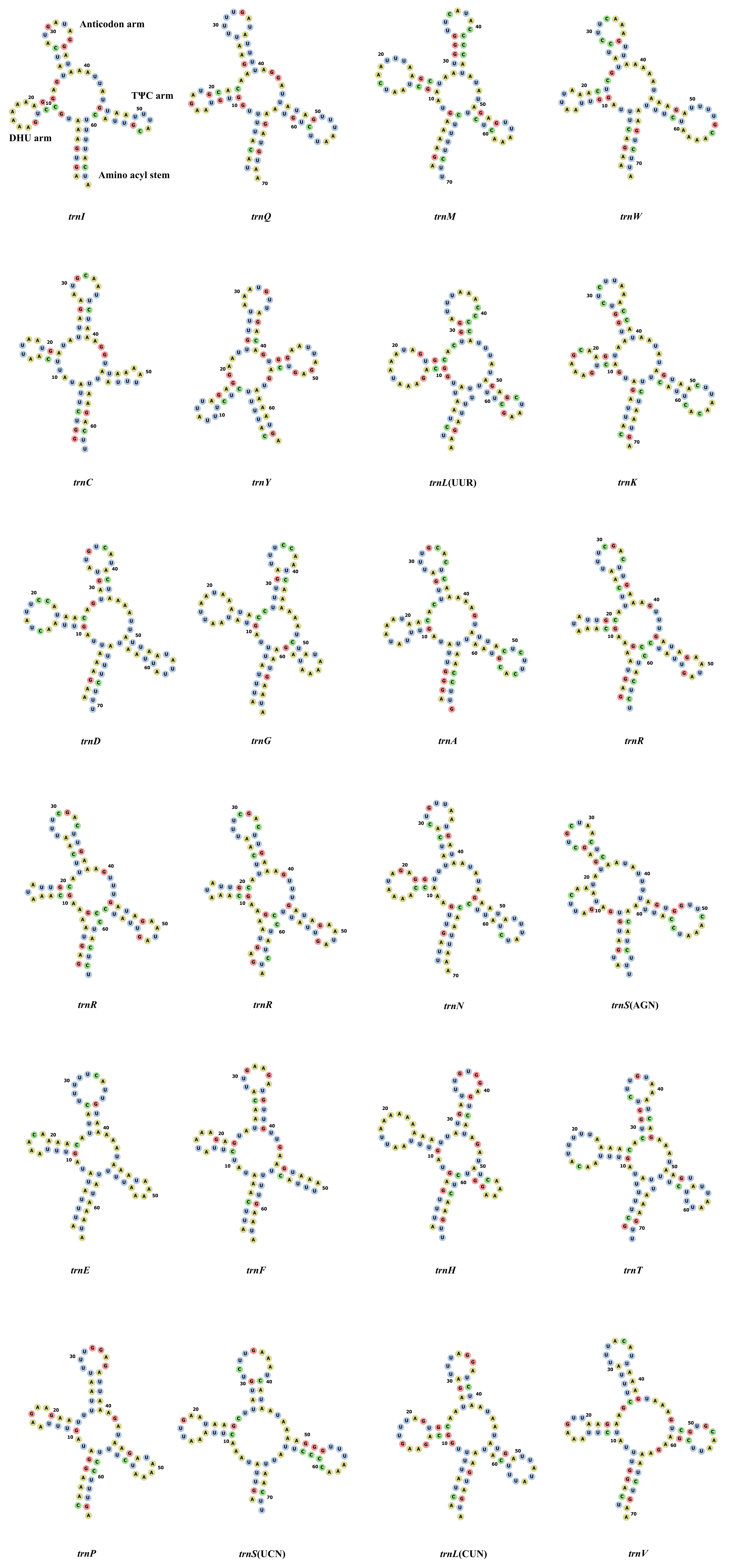

B. *C. straminea*

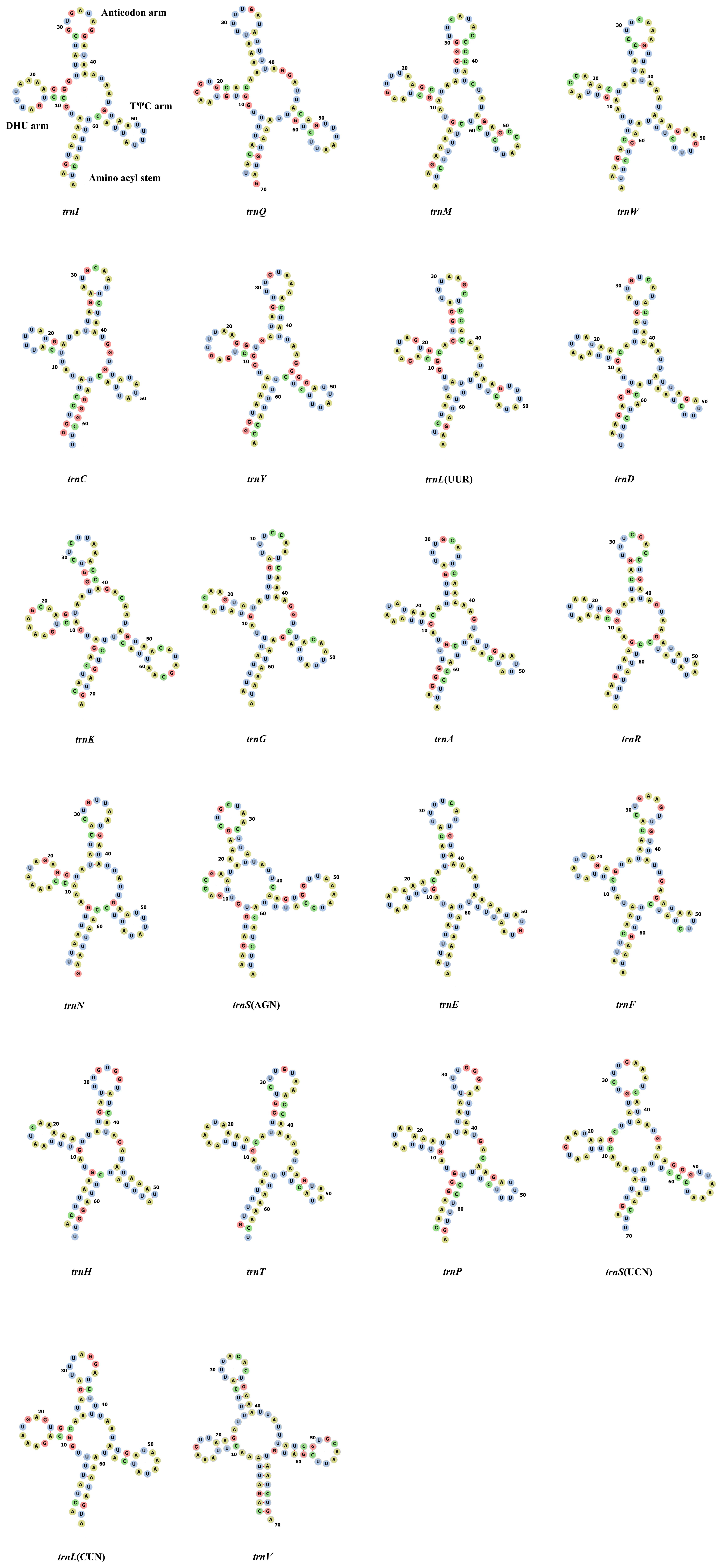

C. *Ha. vitalisi*

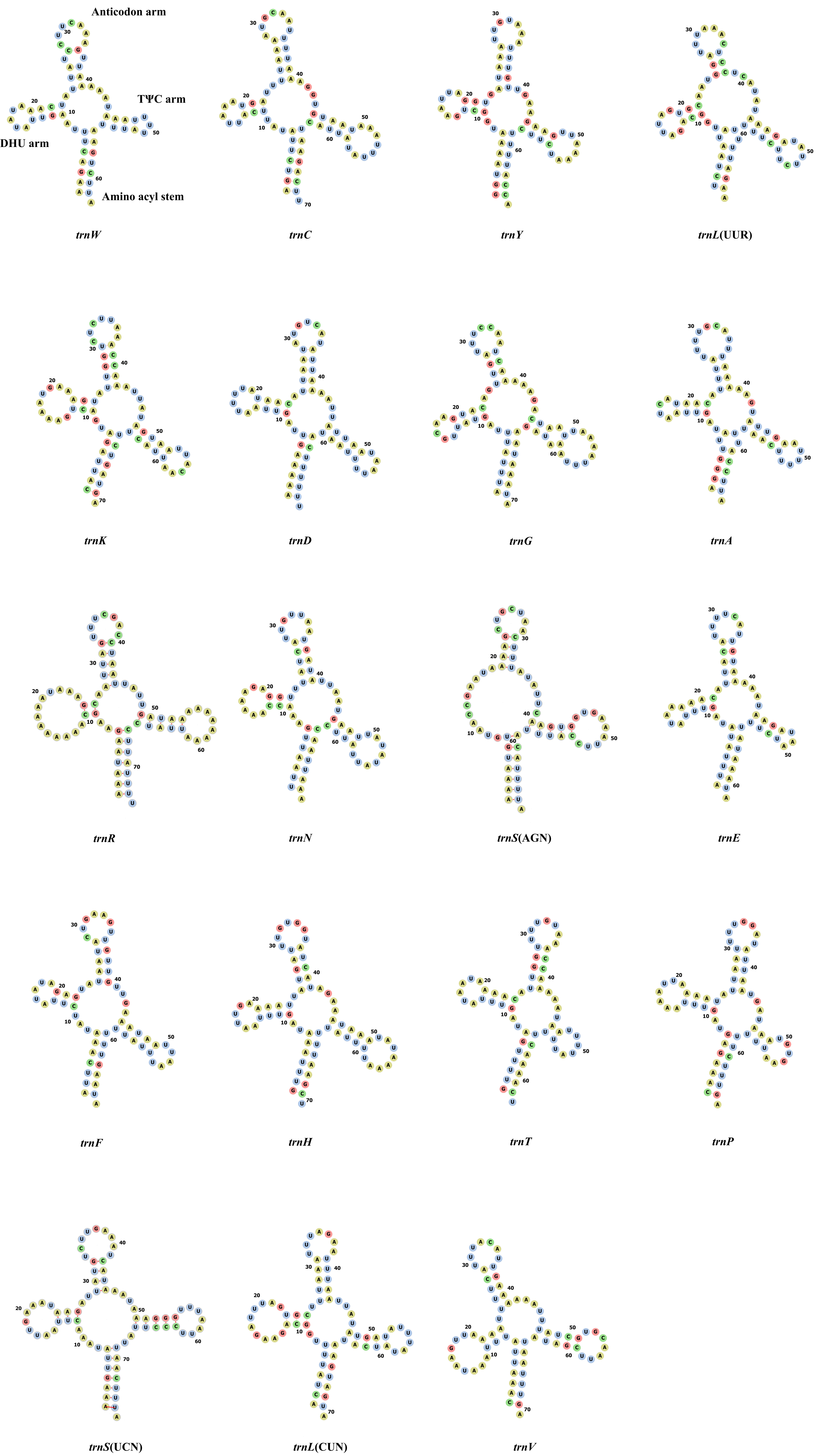

D. *Heterochaeta* sp. JZ-2017

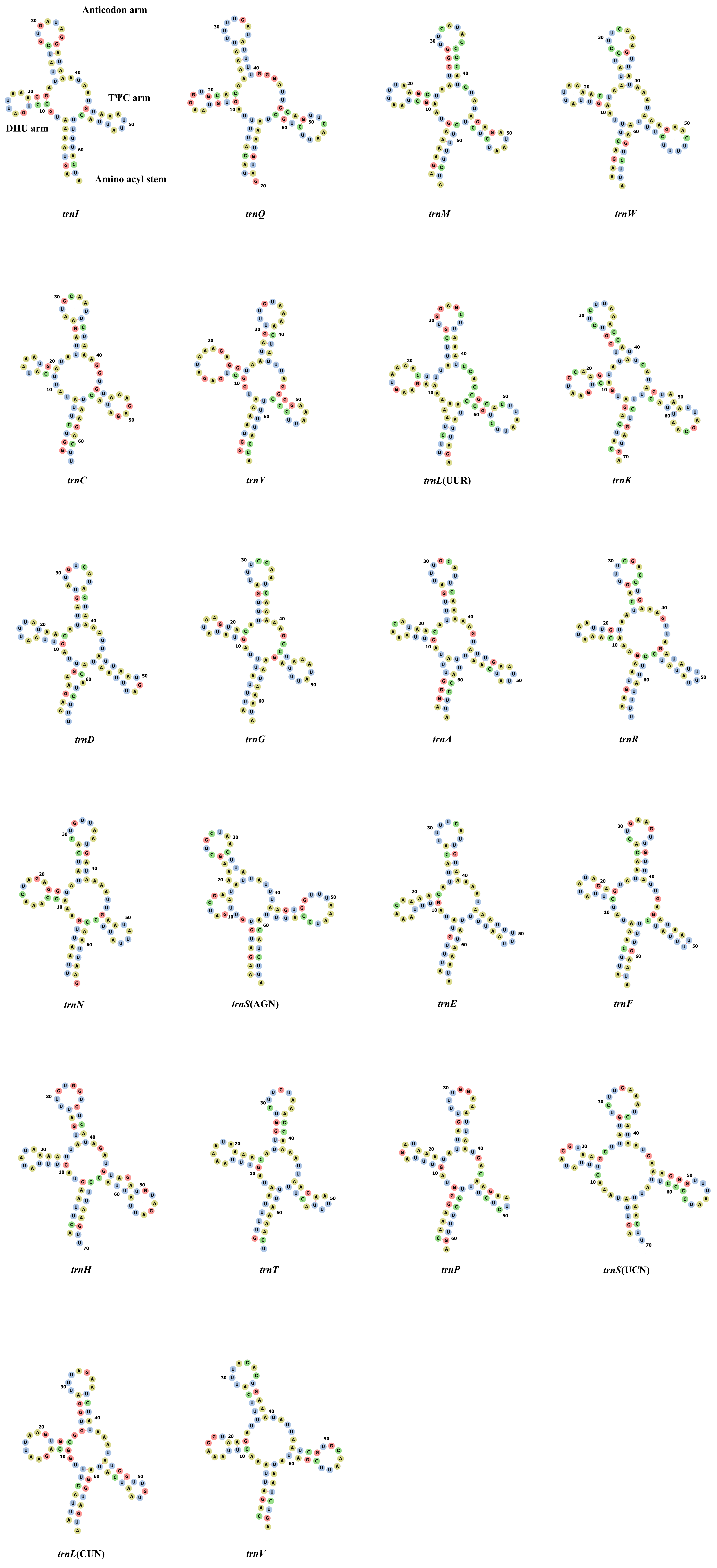

E. *Hu. nada*

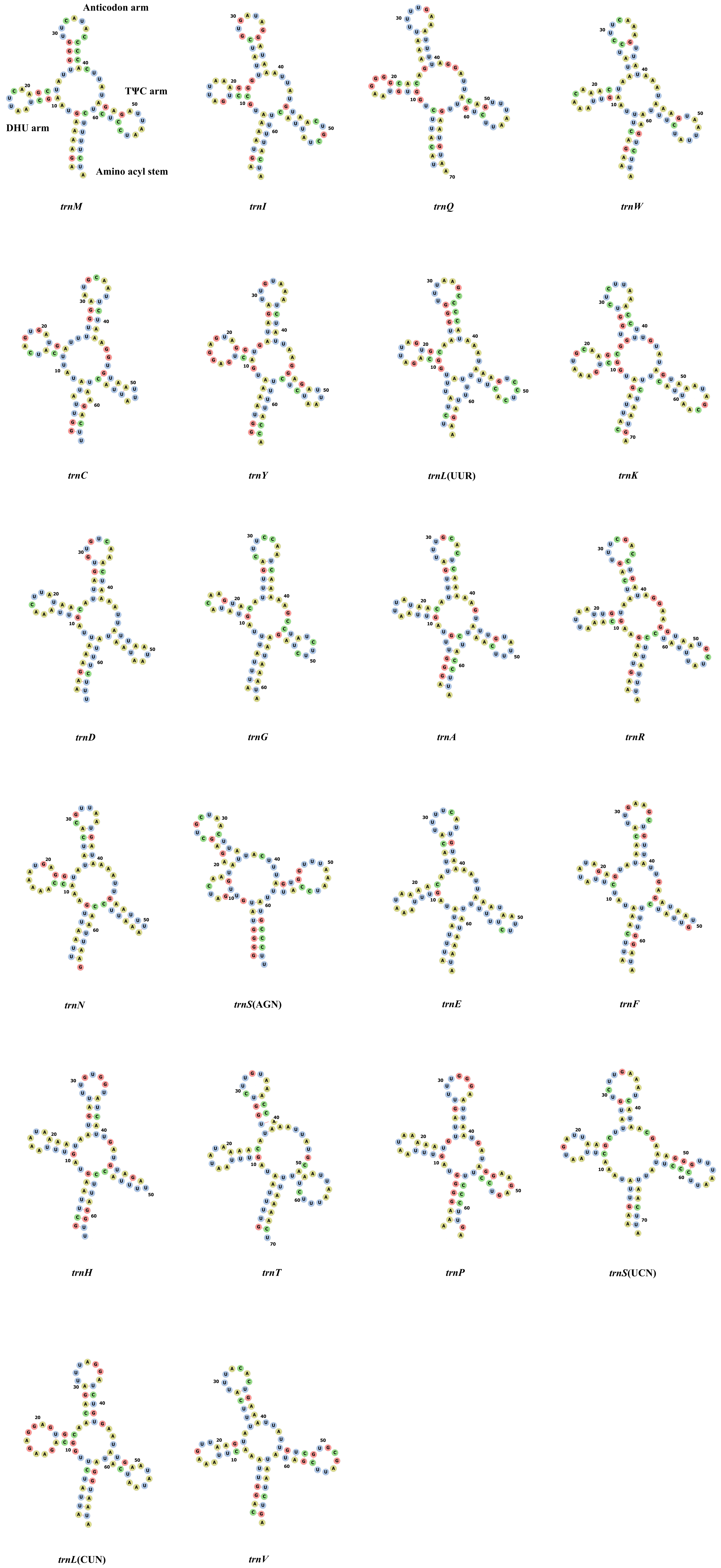
Anticodon arm
TΨC arm
DHU arm
Amino acyl stem
trnM
trnI
trnQ
trnW
trnC
trnY
trnL(UUR)
trnK
trnD
trnG
trnA
trnR
trnN
trnS(AGN)
trnE
trnF
trnH
trnT
trnP
trnS(UCN)
trnL(CUN)
trnV

F. ***P. chlorophaea***

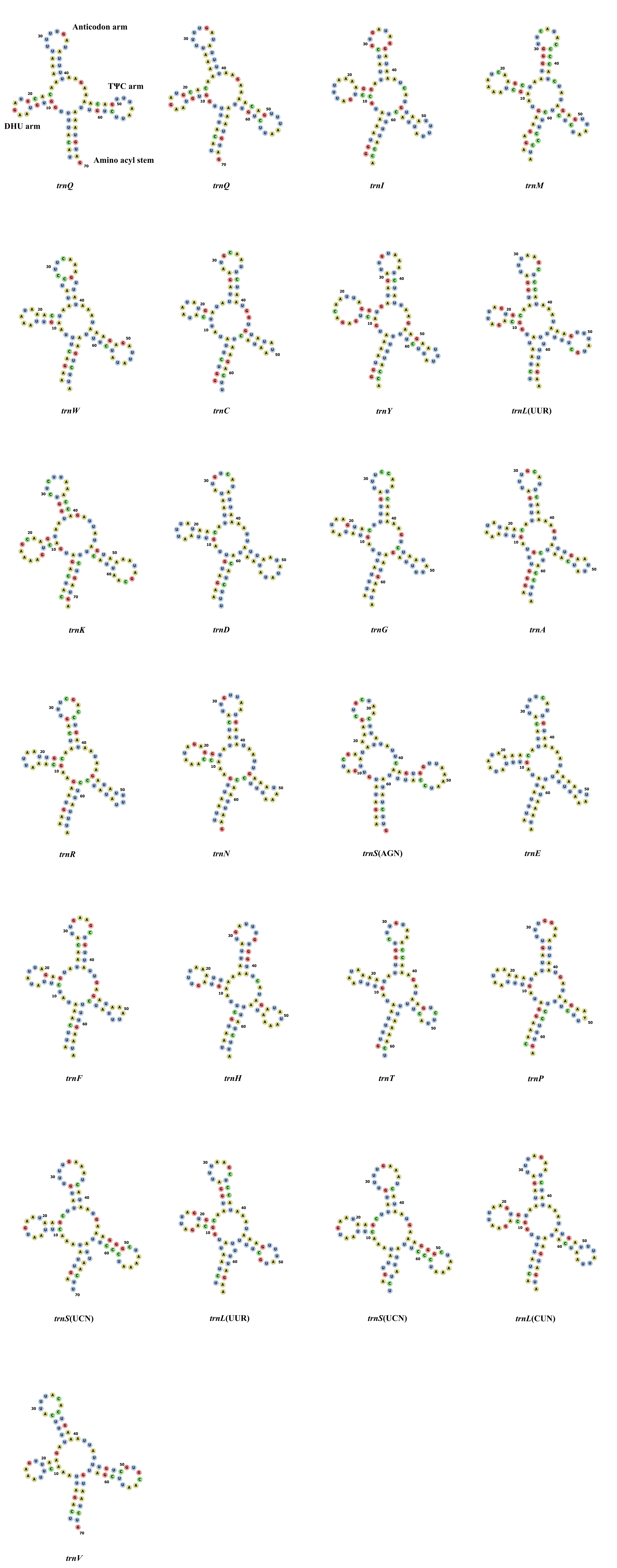

G. *Si. grahami*

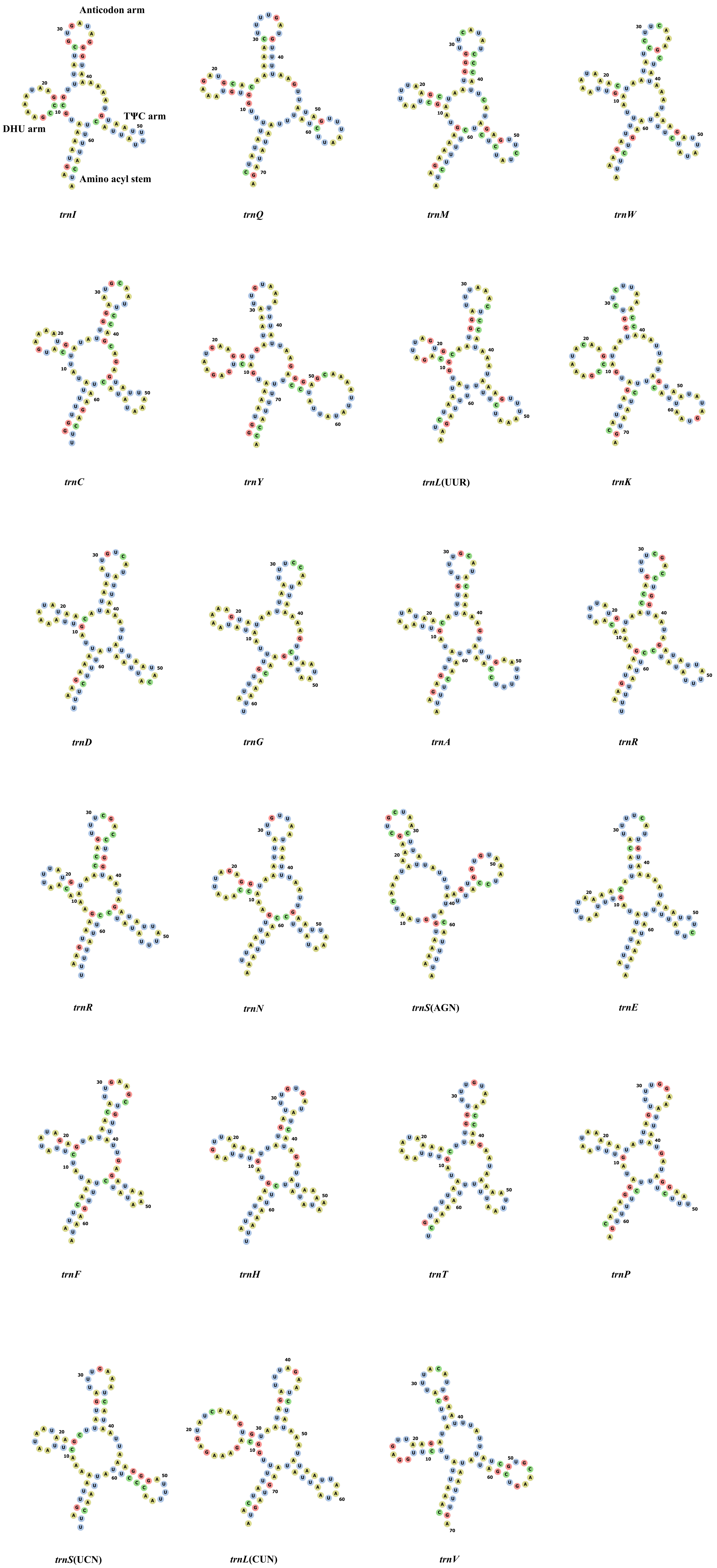

Anticodon arm
DHU arm
TΨC arm
Amino acyl stem
trnI
trnQ
trnM
trnW
trnC
trnY
trnL(UUR)
trnK
trnD
trnG
trnA
trnR
trnR
trnN
trnS(AGN)
trnE
trnF
trnH
trnT
trnP
trnS(UCN)
trnL(CUN)
trnV

H. *Sp. occipitalis*

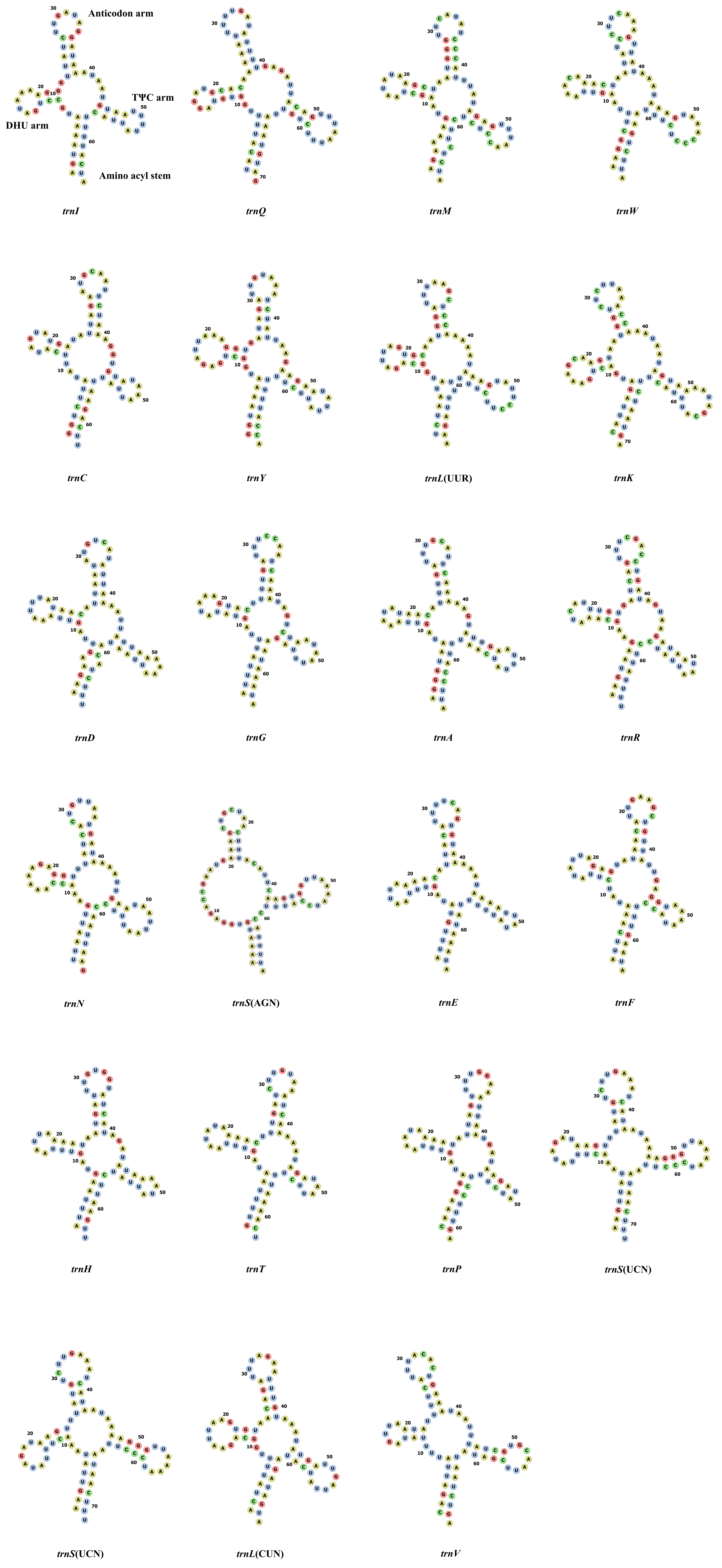

I. *T. milligratulata*

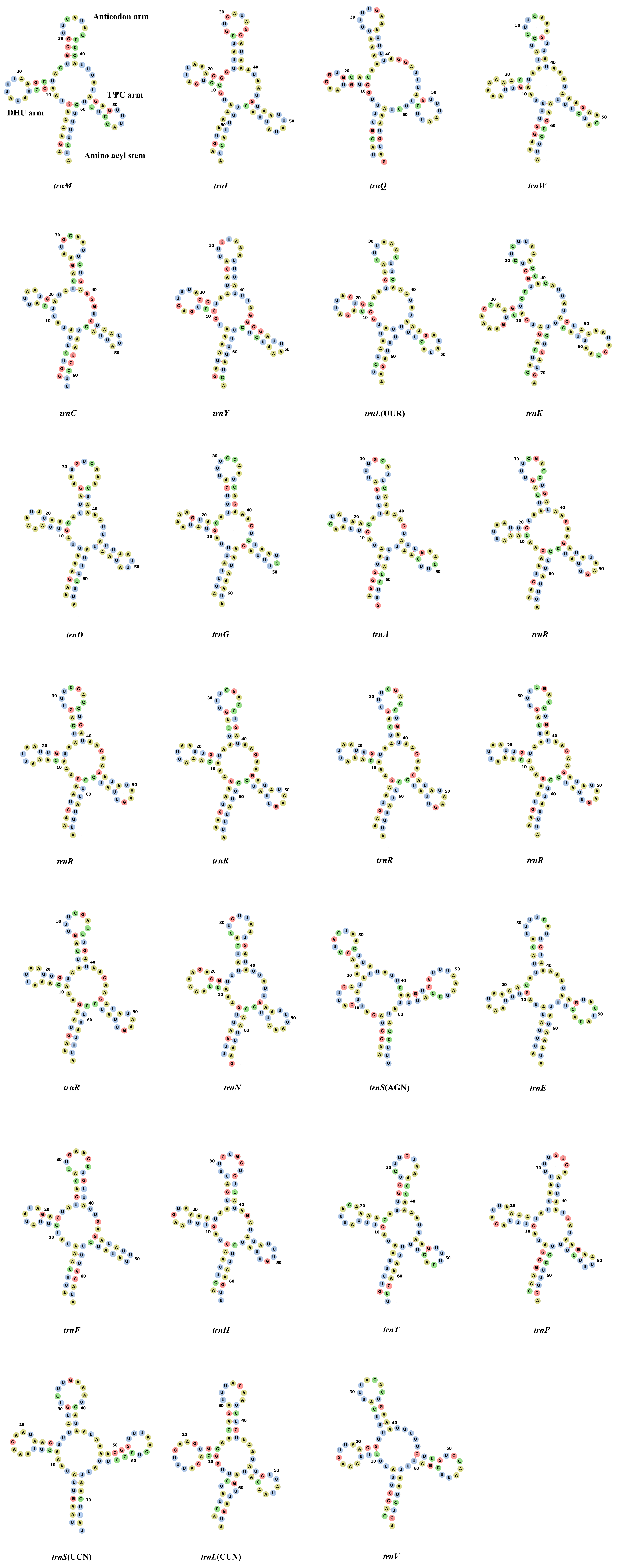

Supplement: Supplementary file 1 [file insects-13-00564-s001.zip › Figure S2. Inferred secondary structures of the tRNA genes in nine mantis mitogenomes.pdf]
