# Supplementary material for: Novel Mitochondrial Gene Rearrangement and Intergenic Regions Exist in the Mitochondrial Genomes from Four Newly Established Families of Praying Mantises (Insecta: Mantodea)
Source: Insects. 2022 Jun 21;13(7):564. doi: 10.3390/insects13070564 (PMC9320148; doi:10.3390/insects13070564)
Supplement: Supplementary file 1 [file insects-13-00564-s001.zip › Table S1.species of mantodea used to construct the phylogenetic relationships along with genbank accession numbers (1).pdf]

Table S1. Species of Mantodea used to construct the phylogenetic relationships along with GenBank accession numbers

Family	Subfamilies	Species	Genome length	GenBank No.	References
Amelidae	/	*Yersinia mexicana*	16,193 bp	MW357303	[7]
Amorphoscelidae	Amorphoscelinae	*Amorphoscelis hainana*	15,792 bp	MW340855	Unpublished
Deroplatyidae	Deroplatyinae	*Deroplatys desiccata*	16,065 bp	KY689113	[10]
Eremiaphilidae	Iridinae	*Schizocephala bicornis*	16,026 bp	KY689135	[10]
Gonypetidae	Gonypetinae	*Humbertiella nada*	15,866 bp	KU201315	[30]
		Humbertiella nada	15,682 bp	KX434857	This study
		Theopompa milligratulata	16,046 bp	KX434849	This study
		Theopompa sp. FY-2016a isolate Hainan	17,370 bp	KU201313	[30]
		Theopompa sp. FY-2016a isolate Yunnan	16,431 bp	KU201314	[30]
Iridopterygidae	Iridopteryginae	*Amantis nawai*	15,684 bp	KY689114	[10]
		Spilomantis occipitalis	15,767 bp	KX434816	This study
		Hapalopeza occipitalis	15,510 bp	KX091854.1	Unpublished
		Iridopterygidae JZ-2017	16,528 bp	KY689120	[10]
Haaniidae	Caliridinae	*Caliris* sp. JZ-2017	15,433 bp	KY689126	[10]
	Haaniinae	*Arria pallida*	16,240 bp	MT594484	Unpublished
		Haania sp. JZ-2017	14,810 bp	KY689130	[10]
		Haania vitalisi	13,628 bp	KX434845	This study
Hymenopodidae	Acromantinae	*Sinomiopteryx grahami*	15,978 bp	KX434860	This study
		Psychomantis borneensis	15,493 bp	MG520077	[66]
		Ambivia undata	15,684 bp	KY689128	[10]
	Hymenopodinae	*Anaxarcha zhengi*	16,620 bp	KU201320	[30]
		Creobroter elongata	15,578 bp	KX091851	Unpublished
		Creobroter gemmatus	15,716 bp	KU201319	[30]
		Creobroter jiangxiensis	15,801 bp	KY689134	[10]
		Creobroter urbanus	15,720 bp	KY689137	[10]
		Odontomantis sp. JZ-2017	15,831 bp	KY689121	[10]
		Theopropus elegans	15,660 bp	KY689125	[10]
	Oxypilinae	*Ceratomantis saussurii*	15,664 bp	KX091850	Unpublished
		Hestiasula sp. FS-2017	15,999 bp	KX091855	Unpublished
		Hestiasula sp. JZ-2017	15,839 bp	KY689115	[10]
		Parablepharis kuhlii (*asiatica*)	16,130 bp	KY689117	[10]
	Phyllothelyinae	*Phyllothelys breve*	15,896 bp	MT024239	[84]
		Phyllothelys shaanxiense	15,586 bp	KX091863	Unpublished
		Phyllothelys sp. 1 JZ-2017	16,467 bp	KY689119	[10]
		Phyllothelys sp. 2 JZ-2017	16,239 bp	KY689129	[10]
	Sibyllinae	*Sibylla pretiosa*	15,829 bp	KY689116	[10]
Leptomantellidae	/	*Leptomantella tonkinae*	15,527 bp	OK480879	Unpublished
	/	*Leptomantella albella*	15,534 bp	KJ463364	[68]
Mantidae	Choeradodinae	*Asiadodis yunnanensis*	15,527 bp	MW357297	[7]
	Hierodulinae	*Hierodula chinensis*	14,882 bp	KY689131	[10]

		Hierodula formosana	16,266 bp	KR703238	[67]
		Hierodula membranacea	16,122 bp	KR703239	Unpublished
		Hierodula membranacea	14,795 bp	KY689112	[10]
		Hierodula patellifera	15,748 bp	KX091856	Unpublished
		Hierodula patellifera	16,999 bp	KX611803	[44]
		Hierodula sp. JYZ-2020	16,748 bp	MW357298	[7]
		Hierodula zhangi	16,235 bp	MW357299	[7]
		Hierodulella sp. JZ-2017	15,477 bp	KY689136	[10]
		Rhombodera brachynota	16,616 bp	KX611802	[44]
		Rhombodera brachynota	16,067 bp	KY689124	[10]
		Rhombodera latipronotum	15,543 bp	KX091864	Unpublished
		Rhombodera longa	15,886 bp	MT110155	[8]
		Rhombodera sp. FY-2017	15,910 bp	KX619654	[44]
		Rhombodera valida	16,308 bp	KX611804	[44]
		Tamolanica tamolana	16,055 bp	DQ241797	[29]
	Mantinae	*Mantis religiosa*	15,534 bp	KU201317	[30]
		Mantis religiosa	15,521 bp	MN356097	[65]
		Statilia maculata	15,775 bp	KX900484	[69]
		Statilia sp. FY-2016a	16,294 bp	KU201316	[30]
	Tenoderinae	*Mesopteryx alata*	18,074 bp	MW357300/KX434813	[7]
		Sphodromantis lineola	15,475 bp	KY689123	[10]
		Tenodera sinensis	15,531 bp	KU201318	[30]
		Tenodera sinensis	15,533 bp	KX673199	[11]
		Tenodera sinensis	15,533 bp	KY689132	[10]
	Vatinae	*Pseudovates chlorophaea*	16,253 bp	KX434838	This study
		Pseudovates peruviana	15,690 bp	MW357301	[7]
		Stagmatoptera biocellata	16,366 bp	MW357302	[7]
Metallyticidae	/	*Metallyticus* sp. JZ-2017	15,056 bp	KY689122	[10]
Nanomantidae	Nanomantinae	*Sceptuchus simplex*	15,548 bp	KY689133	[10]
	Tropidomantinae	*Eomantis yunnanensis*	15,466 bp	KY689138	[10]
		Pliacanthopus bimaculatus	15,941 bp	MT679725	[22]
		Tropidomantis tenera	15,609 bp	KY689127	[10]
Toxoderidae	Heterochaetinae	*Heterochaeta* sp. JZ-2017	15,576 bp	KX434823	This study
	Toxoderinae	*Paratoxodera polyacantha*	15,999 bp	MG049920	[12]
		Stenotoxodera porioni	15,633 bp	KY689118	[10]
		Toxodera hauseri	15,616 bp	KX434837	[12]
Thespidae	Oxyothespinae	*Cheddikulama straminea*	16,236 bp	OM910844	This study
	Thespinae	*Carrikerella* sp.	16,053 bp	OM910846	This study
Blattodea (outgroup)	Corydiidae	*Eupolyphaga sinensis*	15,553 bp	FJ830540	[72]
	Cryptocercidae	*Cryptocercus kyebangensis*	15,720 bp	KP872847	[72]
	Termitidae	*Termes hospes*	16,461 bp	KP091693	[70]
		Macrotermes barneyi	15,940 bp	JX050221	[71]
