# Supplementary material for: Novel Mitochondrial Gene Rearrangement and Intergenic Regions Exist in the Mitochondrial Genomes from Four Newly Established Families of Praying Mantises (Insecta: Mantodea)
Source: Insects. 2022 Jun 21;13(7):564. doi: 10.3390/insects13070564 (PMC9320148; doi:10.3390/insects13070564)
Supplement: Supplementary file 1 [file insects-13-00564-s001.zip › Table S10. Location of features in the mtDNA of Humbertiella nada.pdf]

Table S10. Location of features in the mtDNA of *Humbertiella nada*

Gene	Strand	Position	Length (nuc.)	Anticodon	Start codon	Stop codon	Intergenic nucleotides
trnM	+	1-68	68	CAU			+136
trnI	+	205-270	66	GAU			+9
trnQ	-	280-349	70	UUG			+63
ND2	+	413-1441	1029		ATT	TAA	+18
trnW	+	1460-1526	67	UCA			-8
trnC	-	1519-1583	65	GCA			+1
trnY	-	1585-1649	65	GUA			-1
COX1	+	1649-3184	1536		CTG	TAA	+5
trnL2 (UUR)	+	3190-3254	65	UAA			0
COX2	+	3255-3939	685		ATG	T	0
trnK	+	3940-4010	71	CUU			-1
trnD	+	4010-4074	65	GUC			0
ATP8	+	4075-4236	162		ATT	TAA	-4
ATP6	+	4233-4910	678		ATA	TAA	-1
COX3	+	4910-5697	788		ATG	TA	-1
trnG	+	5697-5758	62	UCC			0
ND3	+	5759-6110	352		ATT	T	0
trnA	+	6111-6174	64	UGC			+4
trnR	+	6179-6247	69	UCG			+13
trnN	+	6261-6325	65	GUU			0
trnS1 (AGN)	+	6326-6396	71	GCU			0
trnE	+	6397-6462	66	UUC			-2
trnF	-	6461-6524	64	GAA			0
ND5	-	6525-8247	1723		ATG	T	0
trnH	-	8248-8310	63	GUG			+10
ND4	-	8321-9658	1338		ATG	TAA	-7
ND4L	-	9652-9933	282		ATG	TAA	+6
trnT	+	9940-10009	70	UGU			0
trnP	-	100010-10073	64	UGG			+3
ND6	+	100077-10583	507		ATT	TAA	-1
Cyt b	+	10583-11725	1143		ATG	TAA	-2
trnS2 (UCN)	+	11724-11795	72	UGA			+17
ND1	-	11813-12745	933		ATG	TAA	+1
trnL1 (CUN)	-	12747-12814	68	UAG			0
16S rRNA	-	12815-14132	1318				0
trnV	-	14133-14201	69	UAC			0
12S rRNA	-	14202-14981	780				0
CR	/	14982-15862	881				
