# Supplementary material for: Novel Mitochondrial Gene Rearrangement and Intergenic Regions Exist in the Mitochondrial Genomes from Four Newly Established Families of Praying Mantises (Insecta: Mantodea)
Source: Insects. 2022 Jun 21;13(7):564. doi: 10.3390/insects13070564 (PMC9320148; doi:10.3390/insects13070564)
Supplement: Supplementary file 1 [file insects-13-00564-s001.zip › Table S11. Location of features in the mtDNA of Carrikerella sp..pdf]

Table S11. Location of features in the mtDNA of *Carrikerella* sp.

Gene	Strand	Position	Length (nuc.)	Anticodon	Start codon	Stop codon	Intergenic nucleotides
trnI	+	1-67	67	GAU			+8
trnQ	-	76-145	70	UUG			+10
trnM	+	156-225	70	CAU			+1
ND2	+	227-1258	1032		ATG	TAA	+40
trnW	+	1299-1370	72	UCA			-8
trnC	-	1363-1425	63	GCA			0
trnY	-	1426-1489	64	GUA			+17
COX1	+	1507-3042	1536		ATG	TAA	+35
trnL2 (UUR)	+	3078-3146	69	UAA			+39
COX2	+	3186-3870	685		ATG	T	+6
trnK	+	3877-3947	71	CUU			-1
trnD	+	3947-4017	71	GUC			+2
ATP8	+	4020-4178	159		ATG	TAA	-7
ATP6	+	4172-4852	681		ATG	TAA	+16
COX3	+	4869-5655	787		ATG	T	0
trnG	+	5656-5722	67	UCC			0
ND3	+	5723-6076	354		ATT	TAA	+36
trnA	+	6113-6181	69	UGC			+1
trnR	+	6183-6248	66	UCG			+19
trnR	+	6268-6333	66	UCG			+19
trnR	+	6353-6418	66	UCG			0
trnN	+	6419-6488	70	GUU			0
trnS1 (AGN)	+	6489-6556	68	GCU			+6
trnE	+	6563-6626	64	UUC			+23
trnF	-	6650-6713	64	GAA			0
ND5	-	6714-8433	1720		ATG	T	+11
trnH	-	8445-8511	67	GUG			+32
ND4	-	8544-9869	1336		ATG	TAG	-7
ND4L	-	9863-10144	282		ATG	TAA	+14
trnT	+	10159-10230	72	UGU			0
trnP	-	10231-10295	65	UGG			+9
ND6	+	10305-10844	540		ATT	TAA	-5
Cyt b	+	10840-11973	1134		ATT	TAA	+4
trnS2 (UCN)	+	11978-12049	72	UGA			+20
ND1	-	12070-13005	936		ATG	TAA	+3
trnL1 (CUN)	-	13009-13077	69	UAG			0
16S rRNA	-	13078-14435	1358				0
trnV	-	14436-14506	71	UAC			0
12S rRNA	-	14507-15334	829				0
CR		15335-16053	719				
