# Supplementary material for: Novel Mitochondrial Gene Rearrangement and Intergenic Regions Exist in the Mitochondrial Genomes from Four Newly Established Families of Praying Mantises (Insecta: Mantodea)
Source: Insects. 2022 Jun 21;13(7):564. doi: 10.3390/insects13070564 (PMC9320148; doi:10.3390/insects13070564)
Supplement: Supplementary file 1 [file insects-13-00564-s001.zip › Table S12. Statistics of TDRs in the CRs of Mantodea mitogenomes..pdf]

Table S12. Statistics of TDRs in the CRs of Mantodea mitogenomes.

Family	Species	Size(bp)	TDRs	Positions	Consensus size (bp)	Copy number	Percent Matches (%)	AT%
Gonypetidae	*Theopompa milligratulata*	1085	1	188-991	130	6.2	97	62.24
			2	188-992	258	3.1	96	62.24
Iridopterygidae	Iridopteryginae JZ-2017	1392	1	43-660	209	3.0	97	81.63
Haaniidae	*Arria pallida*	1289	1	1-373	125	3.0	98	73.73
			2	938-975	15	2.5	83	100.00
Toxoderidae	*Heterochaeta* sp. JZ-2017	820	1	17-56	21	1.9	85	84.84
	Paratoxodera polyacantha	710	1	316-389	31	2.4	93	90.81
