# Supplementary material for: Novel Mitochondrial Gene Rearrangement and Intergenic Regions Exist in the Mitochondrial Genomes from Four Newly Established Families of Praying Mantises (Insecta: Mantodea)
Source: Insects. 2022 Jun 21;13(7):564. doi: 10.3390/insects13070564 (PMC9320148; doi:10.3390/insects13070564)
Supplement: Supplementary file 1 [file insects-13-00564-s001.zip › Table S13. Conserved block sequences (CBSs) in CRs within four families.pdf]

Table S13. Conserved block sequences (CBSs) in CRs within four families.

Family	Subfamily	Species	Conserved region in subfamily	Length (bp)	Conserved region in family	Length (bp)
Gonypetidae	Gonypetinae (Gonypetidae)	*Humbertiella nada*	248-516	268	268-315;424-514	47;90
		Humbertiella nada	262-530	268	282-329;438-528	47;90
		Theopompa sp. FY-2016a isolate Hainan	1181-1453	272	1203-1249;1361-1451	46;90
		Theopompa sp. FY-2016a isolate Yunnan	817-1087	270	839-885;994-1066	46;72
	Iridopteryginae (Iridopterygidae)	*Amantis nawai*	320-549	229	257-302;407-477	45;70
		Spilomantis occipitalis	320-550	230	261-304;407-478	43;71
		Hapalopeza occipitalis	344-574	230	267-308;431-502	41;71
		Iridopteryginae JZ-2017	755-981	226	683-721;841-910	38;69
Haaniidae	Caliridinae	*Caliris sp. JZ-2017*	-	/	314-340;353-393	26;41
	Haaniinae	*Arria pallida*	346-388;622-1027	42;406	695-721;761-802	26;42
		Haania sp. JZ-2017	\	/	\	/
		Haania vitalisi	\	/	\	/
		Sinomiopteryx grahami	238-196;427-838	42;412	506-532;568-610	26;43
Toxoderidae	Toxoderinae	*Paratoxodera polyacantha*	1-49;149-236;253-306;347-596;659-706	49;88;54;250;48	150-171;257-293;366-435;513-566	21;36;69;53
		Stenotoxodera porioni	1-48;150-237;263-320;328-571;633-680	48;88;58;244;48	151-172;267-303;342-411;488-541	21;36;69;53
		Toxodera hauseri	2-49;150-237;264-321;329-573;635-682	48;88;58;245;48	151-172;265-304;343-412;490-543	21;39;69;53
	Oxyothespinae	*Cheddikulama straminea*	-	/	140-161;768-805;858-927;1008-1061	21;37;69;53
