# Supplementary material for: Novel Mitochondrial Gene Rearrangement and Intergenic Regions Exist in the Mitochondrial Genomes from Four Newly Established Families of Praying Mantises (Insecta: Mantodea)
Source: Insects. 2022 Jun 21;13(7):564. doi: 10.3390/insects13070564 (PMC9320148; doi:10.3390/insects13070564)
Supplement: Supplementary file 1 [file insects-13-00564-s001.zip › Table S2. The partition schemes and best-fitting models selected of 13 protein-coding genes.pdf]

Table S2. The partition schemes and best-fitting models selected of 13 protein-coding genes

Subset	Nucleotide sequence alignments	
	Subset partitions	Best model
Partition 1	*ATP6*_gb_codon1, *NAD6*_gb_codon1, *NAD3*_gb_codon1	GTR+I+G
Partition 2	*CYTB*_gb_codon2, *COX3*_gb_codon2, *COX2*_gb_codon2, *ATP6*_gb_codon2, *NAD3*_gb_codon2, *NAD6*_gb_codon2, *NAD2*_gb_codon2	GTR+I+G
Partition 3	*NAD6*_gb_codon3, *ATP6*_gb_codon3, *ATP8*_gb_codon3, *NAD3*_gb_codon3	TIM+G
Partition 4	*ATP8*_gb_codon1, *ATP8*_gb_codon2, *NAD2*_gb_codon1	HKY+I+G
Partition 5	*COX2*_gb_codon1, *COX1*_gb_codon1	GTR+I+G
Partition 6	*COX1*_gb_codon2	TVM+I+G
Partition 7	*COX3*_gb_codon3, *CYTB*_gb_codon3, *COX1*_gb_codon3, *COX2*_gb_codon3	GTR+I+G
Partition 8	*CYTB*_gb_codon1, *COX3*_gb_codon1	GTR+I+G
Partition 9	*NAD1*_gb_codon1	GTR+I+G
Partition 10	*NAD4L*_gb_codon2, *NAD1*_gb_codon2, *NAD5*_gb_codon2, *NAD4*_gb_codon2	GTR+I+G
Partition 11	*NAD4L*_gb_codon3, *NAD1*_gb_codon3	TRN+G
Partition 12	*NAD2*_gb_codon3	HKY+G
Partition 13	*NAD4L*_gb_codon1, *NAD5*_gb_codon1, *NAD4*_gb_codon1	GTR+I+G
Partition 14	*NAD4*_gb_codon3, *NAD5*_gb_codon3	TVN+G
