# Supplementary material for: Novel Mitochondrial Gene Rearrangement and Intergenic Regions Exist in the Mitochondrial Genomes from Four Newly Established Families of Praying Mantises (Insecta: Mantodea)
Source: Insects. 2022 Jun 21;13(7):564. doi: 10.3390/insects13070564 (PMC9320148; doi:10.3390/insects13070564)
Supplement: Supplementary file 1 [file insects-13-00564-s001.zip › Table S3. Location of features in the mtDNA of Sinomiopteryx grahami.pdf]

Table S3. Location of features in the mtDNA of *Sinomiopteryx grahami*

Gene	Strand	Position	Length (nuc.)	Anticodon	Start codon	Stop codon	Intergenic Nucleotides
trnI	+	1-66	66	GAU			+7
trnQ	-	74-145	72	UUG			-2
trnM	+	144-210	67	CAU			0
ND2	+	211-1236	1026		ATG	TAA	-2
trnW	+	1235-1301	67	UCA			-8
trnC	-	1294-1359	66	GCA			+11
trnY	-	1371-1446	76	GUA			+8
COX1	+	1455-2993	1539		ATT	TAA	+3
trnL2 (UUR)	+	2997-3062	66	UAA			+4
COX2	+	3067-3751	685		ATG	T	0
trnK	+	3752-3823	72	CUU			+1
trnD	+	3825-3890	66	GUC			0
ATP8	+	3891-4049	159		ATT	TAA	-4
ATP6	+	4046-4723	678		ATA	TAA	-1
COX3	+	4723-5509	787		ATG	T	0
trnG	+	5510-5571	62	UCC			0
ND3	+	5572-5925	354		ATT	T	+3
trnA	+	5929-5995	67	UGC			+4
trnR	+	6000-6065	66	UCG			+50
trnR	+	6116-6181	66	UCG			+7
trnN	+	6189-6255	67	GUU			0
trnS1 (AGN)	+	6256-6321	66	GCU			0
trnE	+	6322-6387	66	UUC			-2
trnF	-	6386-6448	63	GAA			+1
ND5	-	6450-8168	1719		ATA	TAA	+3
trnH	-	8172-8236	65	GUG			+1
ND4	-	8238-9581	1344		ATT	TAA	-13
ND4L	-	9569-9850	282		ATG	TAA	+3
trnT	+	9854-9918	65	UGU			0
trnP	-	9919-9981	63	UGG			+2
ND6	+	9984-10487	504		ATT	TAA	-1
Cyt b	+	10487-11623	1137		ATG	TAG	-2
trnS2 (UCN)	+	11622-11689	68	UGA			+20
ND1	-	11710-12642	933		ATG	TAA	+1
trnL1 (CUN)	-	12644-12718	75	UAG			-1
16S rRNA	-	12718-14072	1355				+1
trnV	-	14074-14143	70	UAC			-1
12S rRNA	-	14143-14930	788				0
CR	/	14931-15978	1048				
