# Supplementary material for: Novel Mitochondrial Gene Rearrangement and Intergenic Regions Exist in the Mitochondrial Genomes from Four Newly Established Families of Praying Mantises (Insecta: Mantodea)
Source: Insects. 2022 Jun 21;13(7):564. doi: 10.3390/insects13070564 (PMC9320148; doi:10.3390/insects13070564)
Supplement: Supplementary file 1 [file insects-13-00564-s001.zip › Table S4. Location of features in the mtDNA of Haania vitalisi.pdf]

Table S4. Location of features in the mtDNA of *Haania vitalisi*

Gene	Strand	Position	Length (nuc.)	Anticodon	Start codon	Stop codon	Intergenic nucleotides
ND2	+	1-345	345		ATA	TAA	+1
trnW	+	347-409	63	UCA			-8
trnC	-	402-471	70	GCA			0
trnY	-	472-537	66	GUA			+23
COX1	+	561-2096	1536		TTG	TAA	+6
trnL2 (UUR)	+	2103-2169	67	UAA			+37
COX2	+	2207-2891	685		ATG	T	0
trnK	+	2892-2962	71	CUU			-1
trnD	+	2962-3029	68	GUC			0
ATP8	+	3030-3188	159		ATT	TAA	-4
ATP6	+	3185-3856	672		ATA	TAA	-1
COX3	+	3856-4643	788		ATG	TA	-1
trnG	+	4643-4713	71	UCC			0
ND3	+	4714-5067	354		ATC	TAA	+1
trnA	+	5069-5134	66	UGC			-1
trnR	+	5134-5207	74	UCG			+3
trnN	+	5211-5279	69	GUU			0
trnS1 (AGN)	+	5280-5345	66	GCU			0
trnE	+	5346-5407	62	UUC			+5
trnF	-	5413-5479	67	GAA			0
ND5	-	5480-7193	1714		ATT	T	+6
trnH	-	7200-7270	71	GUG			+16
ND4	-	7287-8615	1329		ATG	TAA	-7
ND4L	-	8609-8890	282		ATG	TAA	+5
trnT	+	8896-8958	63	UGU			0
trnP	-	8959-9024	66	UGG			0
ND6	+	9025-9525	501		ATA	TAA	+8
Cyt b	+	9534-10670	1137		ATG	TAA	+9
trnS2 (UCN)	+	10680-10755	76	UGA			+14
ND1	-	10770-11702	933		ATG	TAA	+2
trnL1 (CUN)	-	11705-11775	71	UAG			0
16S rRNA	-	11776-13095	1320				0
trnV	-	13096-13166	71	UAC			0
12S rRNA	-	13167-13628	462				0
