# Supplementary material for: Novel Mitochondrial Gene Rearrangement and Intergenic Regions Exist in the Mitochondrial Genomes from Four Newly Established Families of Praying Mantises (Insecta: Mantodea)
Source: Insects. 2022 Jun 21;13(7):564. doi: 10.3390/insects13070564 (PMC9320148; doi:10.3390/insects13070564)
Supplement: Supplementary file 1 [file insects-13-00564-s001.zip › Table S5. Location of features in the mtDNA of Pseudovates chlorophaea .pdf]

Table S5. Location of features in the mtDNA of *Pseudovates chlorophaea*

Gene	Strand	Position	Length (nuc.)	Anticodon	Start codon	Stop codon	Intergenic nucleotides
trnQ	-	1-70	70	UUG			+235
trnQ	-	306-375	70	UUG			+201
trnI	+	577-643	67	GAU			+17
trnM	+	661-727	67	CAU			+3
ND2	+	731-1757	1027		ATA	T	0
trnW	+	1758-1826	69	UCA			-8
trnC	-	1819-1881	63	GCA			+2
trnY	-	1884-1952	69	GUA			+3
COX1	+	1956-3491	1536		TTG	TAA	+8
trnL2 (UUR)	+	3500-3565	66	UAA			+7
COX2	+	3573-4257	685		ATG	T	0
trnK	+	4258-4329	72	CUU			+4
trnD	+	4334-4400	67	GUC			0
ATP8	+	4401-4559	159		ATT	TAA	-4
ATP6	+	4556-5233	678		ATA	TAA	-1
COX3	+	5233-6024	792		ATA	TAA	+2
trnG	+	6027-6090	64	UCC			0
ND3	+	6091-6444	354		ATT	TAA	+2
trnA	+	6447-6511	65	UGC			+4
trnR	+	6516-6581	66	UCG			+3
trnN	+	6585-6649	65	GUU			0
trnS1 (AGN)	+	6650-6716	67	GCU			0
trnE	+	6717-6782	66	UUC			+1
trnF	-	6784-6848	65	GAA			0
ND5	-	6849-8571	1723		ATT	T	0
trnH	-	8572-8640	69	GUG			0
ND4	-	8641-9979	1339		ATG	T	-7
ND4L	-	9973-10254	282		ATG	TAA	+4
trnT	+	10259-10320	62	UGU			0
trnP	-	10321-10383	63	UGG			0
ND6	+	10384-10887	504		ATA	TAA	-1
Cyt b	+	10887-12023	1137		ATG	TAA	+2
trnS2 (UCN)	+	12026-12095	70	UGA			+388
trnL2 (UUR)	-	12484-12548	65	UAA			+613
trnS2 (UCN)	+	13162-13231	70	UGA			+31
ND1	-	13263-14189	927		ATG	TAA	+1
trnL1 (CUN)	-	14191-14259	69	UAG			0
16S rRNA	-	14260-15575	1316				0
trnV	-	15576-15646	71	UAC			0
12S rRNA	-	15646-16253	608				
