# Supplementary material for: Novel Mitochondrial Gene Rearrangement and Intergenic Regions Exist in the Mitochondrial Genomes from Four Newly Established Families of Praying Mantises (Insecta: Mantodea)
Source: Insects. 2022 Jun 21;13(7):564. doi: 10.3390/insects13070564 (PMC9320148; doi:10.3390/insects13070564)
Supplement: Supplementary file 1 [file insects-13-00564-s001.zip › Table S6. Location of features in the mtDNA of Heterochaeta sp. JZ-2017.pdf]

Table S6. Location of features in the mtDNA of *Heterochaeta* sp. JZ-2017

Gene	Strand	Position	Length (nuc.)	Anticodon	Start codon	Stop codon	Intergenic nucleotides
trnI	+	1-64	64	GAU			+6
trnQ	-	71-140	70	UUG			+2
trnM	+	143-208	66	CAU			0
ND2	+	209-1237	1029		ATA	TAA	+5
trnW	+	1243-1309	67	UCA			-8
trnC	-	1302-1364	63	GCA			0
trnY	-	1365-1430	66	GUA			+3
COX1	+	1434-2969	1536		TTG	TAA	0
trnL2 (UUR)	+	2970-3038	69	UAA			+2
COX2	+	3041-3727	687		ATG	TAA	+15
trnK	+	3743-3813	71	CUU			+1
trnD	+	3815-3880	66	GUC			0
ATP8	+	3881-4039	159		ATT	TAA	-4
ATP6	+	4036-4713	678		ATA	TAA	+5
COX3	+	4719-5513	795		ATT	TAA	+3
trnG	+	5517-5579	63	UCC			0
ND3	+	5580-5933	354		ATC	TAA	+7
trnA	+	5941-6005	65	UGC			+4
trnR	+	6010-6074	65	UCG			+2
trnN	+	6077-6141	65	GUU			0
trnS1 (AGN)	+	6142-6208	67	GCU			0
trnE	+	6209-6273	65	UUC			0
trnF	-	6274-6337	64	GAA			0
ND5	-	6338-8057	1720		ATG	T	0
trnH	-	8058-8127	70	GUG			0
ND4	-	8128-9465	1338		ATG	TAA	-7
ND4L	-	9459-9740	282		ATG	TAA	+3
trnT	+	9744-9808	65	UGU			0
trnP	-	9809-9870	62	UGG			+2
ND6	+	9873-10376	504		ATT	TAA	-1
Cyt b	+	10376-11512	1137		ATG	TAA	+14
trnS2 (UCN)	+	11527-11596	70	UGA			+16
ND1	-	11613-12545	933		ATG	TAG	+1
trnL1 (CUN)	-	12547-12613	67	UAG			0
16S rRNA	-	12614-13926	1313				0
trnV	-	13927-13995	69	UAC			0
12S rRNA	-	13996-14756	761				0
CR		14757-15576	820				
