# Supplementary material for: Novel Mitochondrial Gene Rearrangement and Intergenic Regions Exist in the Mitochondrial Genomes from Four Newly Established Families of Praying Mantises (Insecta: Mantodea)
Source: Insects. 2022 Jun 21;13(7):564. doi: 10.3390/insects13070564 (PMC9320148; doi:10.3390/insects13070564)
Supplement: Supplementary file 1 [file insects-13-00564-s001.zip › Table S7. Location of features in the mtDNA of Spilomantis occipitalis.pdf]

Table S7. Location of features in the mtDNA of *Spilomantis occipitalis*

Gene	Strand	Position	Length (nuc.)	Anticodon	Start codon	Stop codon	Intergenic nucleotides
trnI	+	1-65	65	GAU			-3
trnQ	-	63-133	71	UUG			-1
trnM	+	133-199	67	CAU			0
ND2	+	200-1228	1029		ATG	TAA	-2
trnW	+	1227-1294	68	UCA			-8
trnC	-	1287-1349	63	GCA			+5
trnY	-	1355-1423	69	GUA			0
COX1	+	1424-2959	1536		TTG	TAA	+3
trnL2 (UUR)	+	2963-3029	67	UAA			+1
COX2	+	3031-3715	685		ATG	T	0
trnK	+	3716-3786	71	CUU			+1
trnD	+	3788-3854	67	GUC			0
ATP8	+	3855-4013	159		ATT	TAA	-4
ATP6	+	4010-4687	678		ATA	TAA	-1
COX3	+	4687-5473	787		ATG	T	0
trnG	+	5474-5537	64	UCC			0
ND3	+	5538-5891	354		ATT	TAA	+1
trnA	+	5893-5958	66	UGC			+4
trnR	+	5963-6028	66	UCG			+1
trnN	+	6030-6096	67	GUU			0
trnS1 (AGN)	+	6097-6163	67	GCU			0
trnE	+	6164-6228	65	UUC			-2
trnF	-	6227-6290	64	GAA			0
ND5	-	6291-8010	1720		ATA	T	+3
trnH	-	8014-8077	64	GUG			0
ND4	-	8078-9415	1338		ATG	TAA	-7
ND4L	-	9409-9690	282		ATG	TAA	+4
trnT	+	9695-9757	63	UGU			+96
trnP	-	9854-9915	62	UGG			+2
ND6	+	9918-10427	510		ATT	TAA	-1
Cyt b	+	10427-11561	1135		ATG	T	0
trnS2 (UCN)	+	11562-11633	72	UGA			+237
trnS2 (UCN)	+	11871-11942	72	UGA			+24
ND1	-	11967-12900	934		ATG	T	+1
trnL1 (CUN)	-	12902-12969	68	UAG			0
16S rRNA	-	12970-14267	1298				0
trnV	-	14268-14336	69	UAC			0
12S rRNA	-	14337-15094	758				0
CR		15095-15767	673				
