# Supplementary material for: Novel Mitochondrial Gene Rearrangement and Intergenic Regions Exist in the Mitochondrial Genomes from Four Newly Established Families of Praying Mantises (Insecta: Mantodea)
Source: Insects. 2022 Jun 21;13(7):564. doi: 10.3390/insects13070564 (PMC9320148; doi:10.3390/insects13070564)
Supplement: Supplementary file 1 [file insects-13-00564-s001.zip › Table S8. Location of features in the mtDNA of Cheddikulama straminea.pdf]

Table S8. Location of features in the mtDNA of *Cheddikulama straminea*

Gene	Strand	Position	Length (nuc.)	Anticodon	Start codon	Stop codon	Intergenic nucleotides
trnI	+	1-67	67	GAU			+24
trnQ	-	92-161	70	UUG			+7
trnM	+	169-236	68	CAU			0
ND2	+	237-1268	1032		ATG	TAA	-2
trnW	+	1267-1332	66	UCA			+22
trnC	-	1355-1417	63	GCA			0
trnY	-	1418-1483	66	GUA			+28
COX1	+	1512-3053	1542		ATT	TAA	+15
trnL2 (UUR)	+	3069-3133	65	UAA			+13
COX2	+	3147-3833	687		ATG	TAA	+50
trnD	+	3884-3949	66	GUC			+6
trnK	+	3956-4027	72	CUU			+68
ATP8	+	4096-4254	159		ATC	TAA	-7
ATP6	+	4248-4928	681		ATG	TAA	-1
COX3	+	4928-5716	789		ATG	TAA	+1
trnG	+	5718-5782	65	UCC			0
ND3	+	5783-6136	354		ATG	TAA	+17
trnA	+	6154-6218	65	UGC			+4
trnR	+	6223-6288	66	UCG			+3
trnN	+	6292-6357	66	GUU			0
trnS1 (AGN)	+	6358-6424	67	GCU			0
trnE	+	6425-6491	67	UUC			0
trnF	-	6492-6556	65	GAA			0
ND5	-	6557-8276	1720		ATA	T	+3
trnH	-	8280-8344	65	GUG			+3
ND4	-	8348-9685	1338		ATG	TAA	-7
ND4L	-	9679-9960	282		ATG	TAA	+4
trnT	+	9965-10028	64	UGU			0
trnP	-	10029-10092	64	UGG			+2
ND6	+	10095-10598	504		ATT	TAA	-1
Cyt b	+	10598-11734	1137		ATG	TAA	+10
trnS2 (UCN)	+	11745-11814	70	UGA			+21
ND1	-	11836-12771	936		ATG	TAA	+1
trnL1 (CUN)	-	12773-12839	67	UAG			0
16S rRNA	-	12840-14158	1319				+1
trnV	-	14160-14229	70	UAC			-1
12S rRNA	-	14229-15015	787				0
CR		15016-16236	1221				
