# Supplementary material for: Novel Mitochondrial Gene Rearrangement and Intergenic Regions Exist in the Mitochondrial Genomes from Four Newly Established Families of Praying Mantises (Insecta: Mantodea)
Source: Insects. 2022 Jun 21;13(7):564. doi: 10.3390/insects13070564 (PMC9320148; doi:10.3390/insects13070564)
Supplement: Supplementary file 1 [file insects-13-00564-s001.zip › Table S9. Location of features in the mtDNA of Theopompa milligratulata.pdf]

Table S9. Location of features in the mtDNA of *Theopompa milligratulata*

Gene	Strand	Position	Length (nuc.)	Anticodon	Start codon	Stop codon	Intergenic nucleotides
trnM	+	1-67	67	CAU			0
CR	/	68-1152	1085				0
trnI	+	1153-1217	65	GAU			0
trnQ	-	1218-1286	69	UUG			+59
ND2	+	1346-2374	1029		ATT	TAA	+3
trnW	+	2378-2442	65	UCA			-8
trnC	-	2435-2496	62	GCA			0
trnY	-	2497-2561	65	GUA			-1
COX1	+	2561-4096	1536		CTG	TAA	+6
trnL2 (UUR)	+	4103-4166	64	UAA			+2
COX2	+	4169-4853	685		ATG	T	0
trnK	+	4854-4925	72	CUU			0
trnD	+	4926-4989	64	GUC			0
ATP8	+	4990-5148	159		ATT	TAA	-4
ATP6	+	5145-5822	678		ATA	TAA	-1
COX3	+	5822-6609	788		ATG	TA	-1
trnG	+	6609-6670	62	UCC			0
ND3	+	6671-7022	352		ATC	T	0
trnA	+	7023-7086	64	UGC			+4
trnR	+	7091-7155	65	UCG			+28
trnR	+	7184-7249	66	UCG			+28
trnR	+	7278-7343	66	UCG			+28
trnR	+	7372-7437	66	UCG			+28
trnR	+	7466-7531	66	UCG			+28
trnR	+	7560-7625	66	UCG			+21
trnN	+	7647-7711	65	GUU			0
trnS1 (AGN)	+	7712-7778	67	GCU			0
trnE	+	7779-7843	65	UUC			+1
trnF	-	7845-7908	64	GAA			+1
ND5	-	7910-9632	1723		ATG	T	0
trnH	-	9633-9695	63	GUG			+9
ND4	-	9705-11042	1338		ATG	TAA	-7
ND4L	-	11036-11317	282		ATG	TAA	+4
trnT	+	11322-11385	64	UGU			0
trnP	-	11386-11449	64	UGG			+2
ND6	+	11452-11955	504		ATC	TAA	-1
Cyt b	+	11955-13094	1140		ATG	TAA	-3
trnS2 (UCN)	+	13092-13165	74	UGA			+17
ND1	-	13183-14115	933		ATG	TAA	+1
trnL1 (CUN)	-	14117-14182	66	UAG			0
16S rRNA	-	14183-15498	1316				0

trnV	-	15499-15567	69	UAC	0
12S rRNA	-	15568-16046	479		0
