# Supplementary figures and images for: Novel Mitochondrial Gene Rearrangement and Intergenic Regions Exist in the Mitochondrial Genomes from Four Newly Established Families of Praying Mantises (Insecta: Mantodea)
Source: Insects. 2022 Jun 21;13(7):564. doi: 10.3390/insects13070564 (PMC9320148; doi:10.3390/insects13070564)

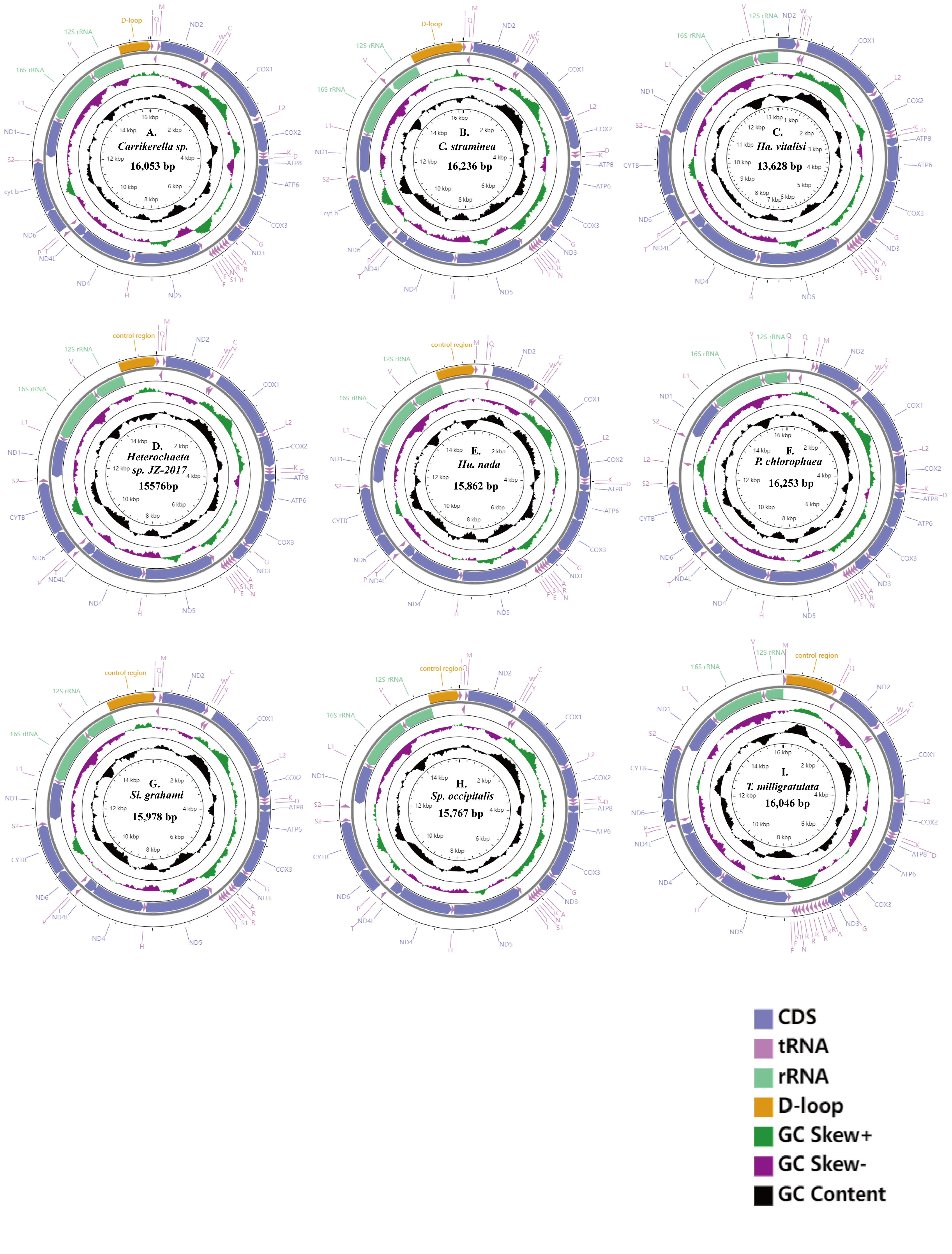

Supplement: Supplementary file 1 [file insects-13-00564-s001.zip › Figure S1. Circular visualization and organization of the complete mitogenome..png]

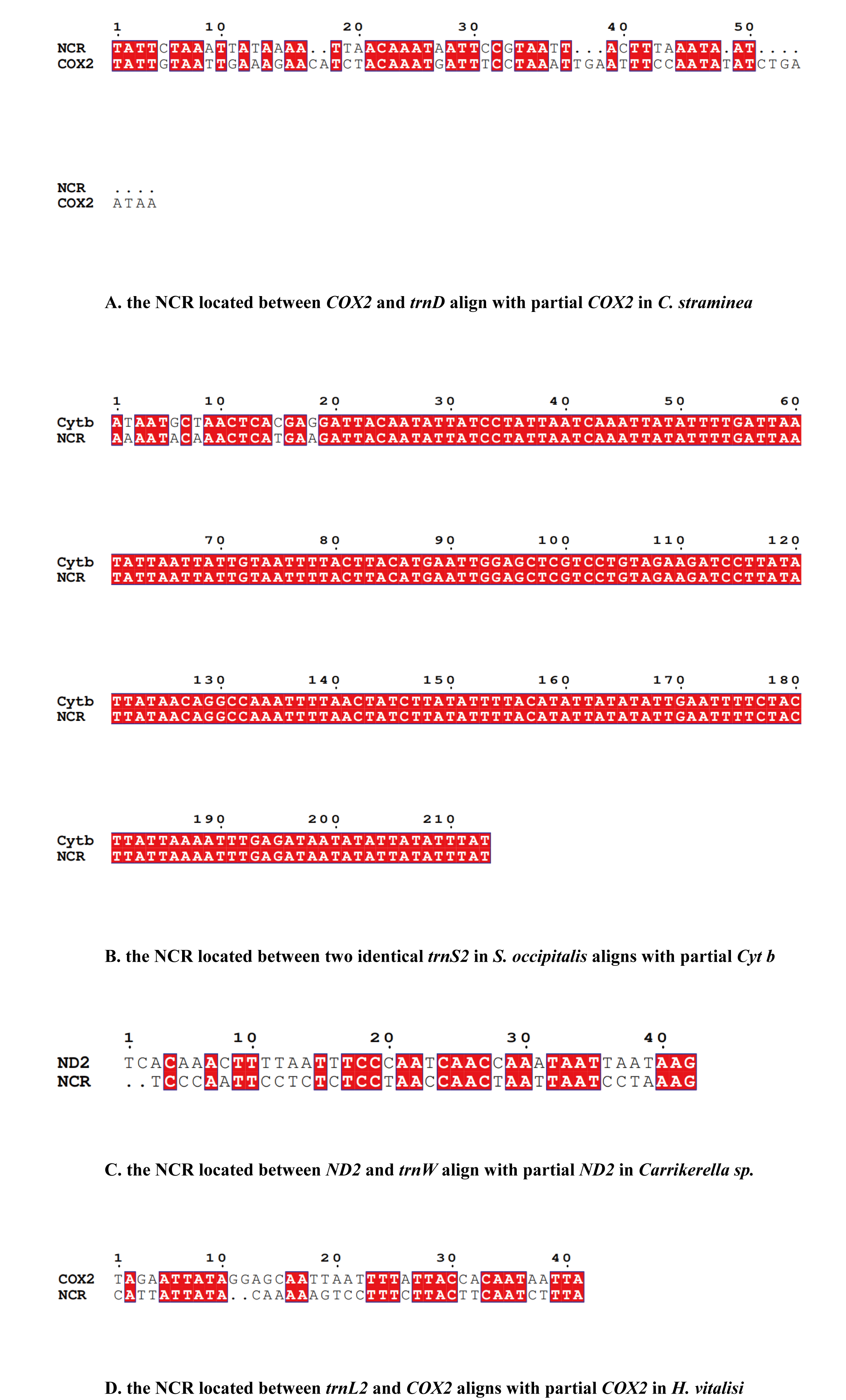

Supplement: Supplementary file 1 [file insects-13-00564-s001.zip › Figure S4. The results of alignment between NCR and adjacent genes.png]

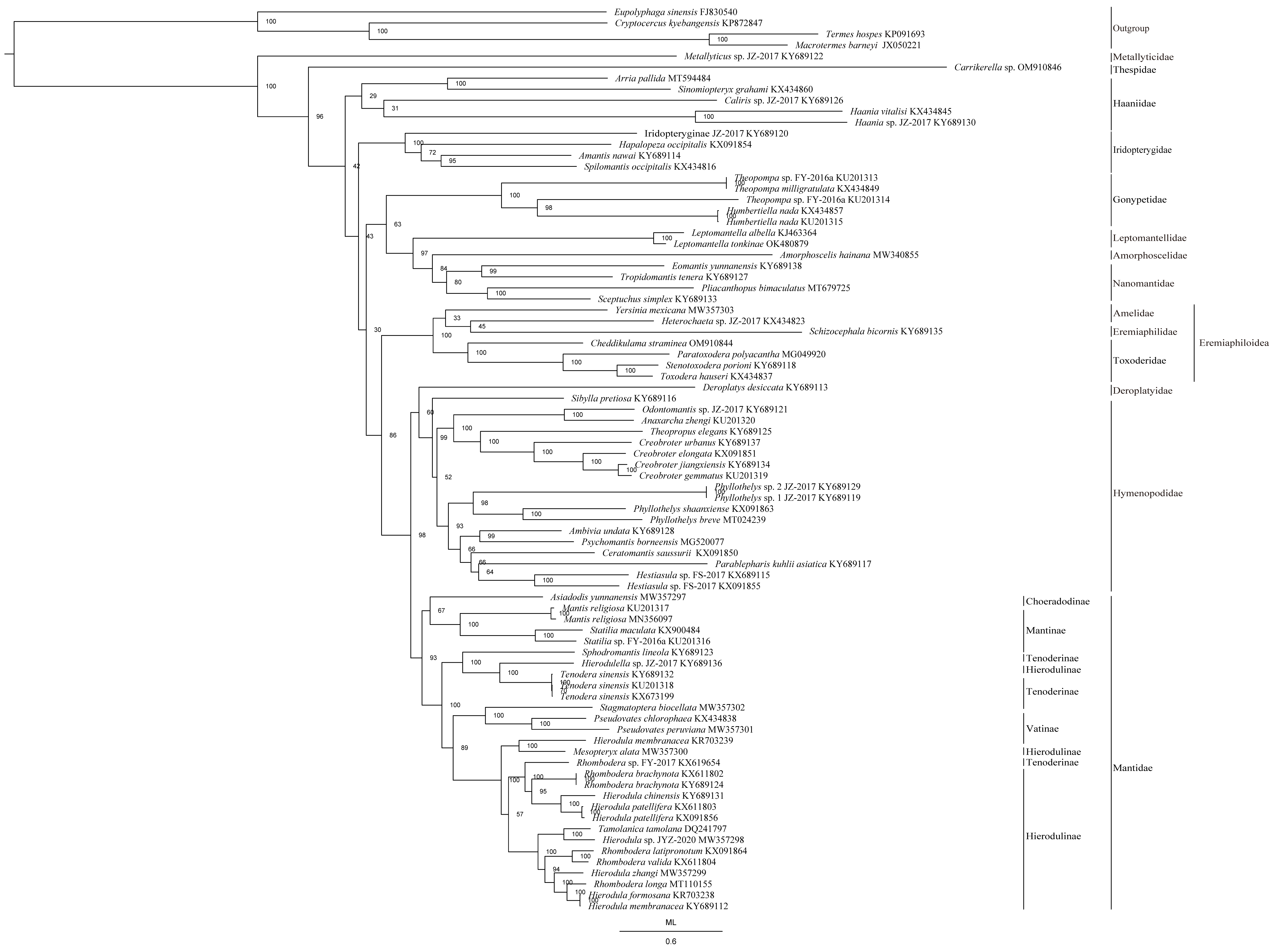

Supplement: Supplementary file 1 [file insects-13-00564-s001.zip › Figure S5. The phylogenetic tree using maximum likelihood (ML) methods.png]
